# Psychological impact of high-quality nursing care on patients with esophageal cancer during perioperative period: A protocol of systematic review

**DOI:** 10.1097/MD.0000000000022270

**Published:** 2020-10-23

**Authors:** Xiu-yu Liu, Chuan-hua Jiao, Dan Zhao, Yan Chen, Hong-mei Zhang

**Affiliations:** a Department of Gastroenterology; b Department of Dermatology, The Second Affiliated Hospital of Mudanjiang Medical University, Mudanjiang, China.

**Keywords:** anxiety, depression, esophageal cancer, high-quality nursing care, perioperative period

## Abstract

**Background:**

This study is designed to systematically assess the psychological impact of high-quality nursing care (HQNC) on patients with esophageal cancer during perioperative period (ECPP).

**Methods:**

Several electronic databases will be searched to collect randomized controlled trials (RCTs) or case-control studies (CCSs) on HQNC in the management of ECPP from inception to present: Cochrane Library, PUBMED, EMBASE, SinoMed, Web of Science, WANGFANG, and China National Knowledge Infrastructure. We will not apply any language limitation to all literature searches. Two authors will independently perform literature selection, data extraction and literature quality evaluation. All disagreements will be resolved by a third author through discussion. Cochrane risk of bias tool will be employed to assess trial quality, and RevMan 5.3 software will be utilized to carry out statistical analysis.

**Results:**

This study will summarize the current evidence to appraise of the psychological impact of HQNC in the management of ECPP.

**Conclusion:**

The findings of this study may help to explicit whether HQNC is effective on psychological problem in ECPP. It will also provide scientific evidence for the clinical practice and future researches.

**Study registration:**

INPLASY202080071.

## 1 Introduction

Esophageal cancer (EC) is one of the most aggressive and malignant diseases worldwide.^[1–4]^ It is also one of the most leading causes of mortality, and represents 5.3% of all cancer-related deaths.^[5]^ In China, it is reported that about 283,433 people died of EC in 2018, which accounts for 9% of total cancer mortality.^[6]^ It mainly manifests as difficulty swallowing, chest pain, worsening indigestion or heartburn, and coughing or hoarseness,^[7,8]^ which leads to very poor quality of life in patients with EC,^[9,10]^ Esophagectomy is the most common treatment for EC.^[11–13]^ Patients with EC also experience a variety of disorders, such as psychological problem (including depression, anxiety, and stress).^[14–17]^ Studies report that high-quality nursing care (HQNC) can benefit psychological disorder in patients with EC during perioperative period (ECPP).^[18–21]^ However, no systematic review specifically investigates the psychological impact of HQNC in patients with ECPP.

## 2 Methods and analysis

### 2.1 Study registration

The present protocol has been registered with INPLASY202080071. We report this study following the guidelines of Preferred Reporting Items for Systematic review and Meta-Analysis (PRISMA) Protocols.^[22,23]^

### 2.2 Eligibility criteria

#### 2.2.1 Type of studies

All randomized controlled trials (RCTs) or case-control studies (CCSs) that appraise the psychological impact of HQNC in the management of ECPP without language and publication status limitations. We will exclude any other studies, such as non-clinical studies, and uncontrolled studies.

#### 2.2.2 Type of participants

All patients with ECPP who were diagnosed as psychological disorder (such as depression and anxiety) will be included in this study, regardless the ethnicity, sex, age, and economic status.

#### 2.2.3 Type of interventions

In the experimental group, all types of HQNC were utilized for the management of psychological disorder in patients with ECPP.

In the control group, any intervention for the management of psychological condition in patients with ECPP will be included. However, we will exclude comparators involved in any forms of HQNC.

#### 2.2.4 Type of outcome measurements

Outcome measurements are depression (as assessed by related scales, such as Major Depression Inventory), anxiety (as appraised by associated scales, such as Generalized Anxiety Disorder 7-item), stress (as measured by relevant tools, such as Acute Stress Disorder Scale), quality of life (as tested by connected scales, such as The Brunnsviken Brief Quality of Life Scale), and any adverse events.

### 2.3 Data sources and search strategy

The following electronic databases will be searched from inception to the present: Cochrane Library, PUBMED, EMBASE, SinoMed, Web of Science, WANGFANG, and China National Knowledge Infrastructure. No language and publication status limitations will be applied to search all literature sources. The detailed search strategy for Cochrane Library is presented in Table 1. We will adapt similar search strategies for other electronic databases.

**Table 1:**
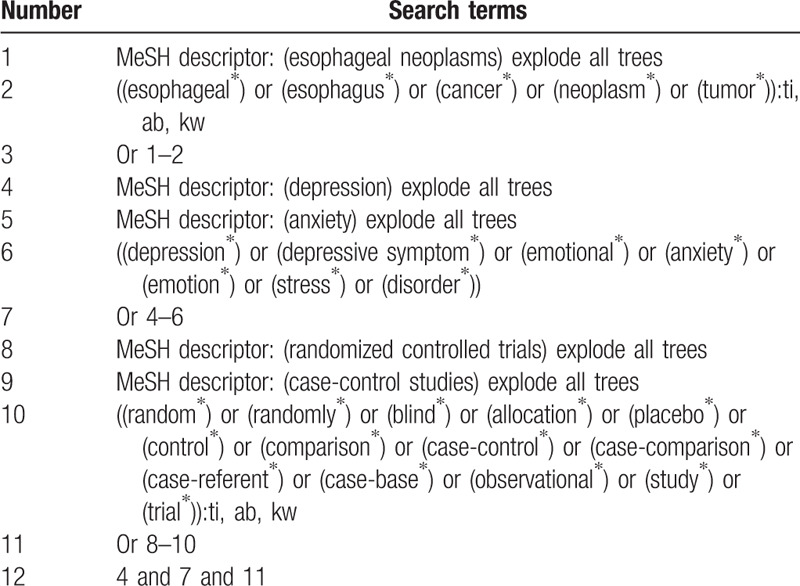
Search strategy for Cochrane Library.

In addition, we will search unpublished postgraduate papers in Chinese databases, abstracts of scientific conferences/symposia, and reference lists of included trials.

## 3 Study selection and data management

### 3.1 Study selection

The whole process of study selection will be carried out by 2 independent authors in according with the previously defined eligibility criteria. First, titles/abstracts of all searched studies will be scanned, and all duplicated and unrelated studies will be removed. Second, full-texts of all remaining potential trials will be carefully read based on all inclusion criteria. Any divergences will be solved through discussion or consultation with a third author. A PRISMA flow chart will be utilized to elaborate the selection procedures of eligible literatures.

### 3.2 Data extraction

Data will be extracted according to the previously designed standardized data collection form by our review team, which will be piloted calibration through at least 3 trials. Two authors will independently extract all essential data from the included trials. Any different opinions will be worked out by discussion with a third author. The extracted data includes study information (such as title, first author, and year of publication), characteristics of population (such as age, gender, and eligibility criteria), study setting, study methods, sample size, details of intervention and control condition, outcome indicators, adverse events, results, findings, follow-up details, and supported findings.

### 3.3 Dealing with missing information

If there is unclear or missing data, original corresponding authors will be contacted to request such information. If this data can not be achieved, we will only analyze available data, and will discuss its potential affects as a limitation.

## 4 Assessment of risk of bias for included trials

The methodological quality of all eligible RCTs will be assessed based on the guideline of Cochrane Risk of Bias Tool,^[24]^ and that of all CCSs will be appraised using The Newcastle-Ottawa Scale^[25]^ by 2 independent authors. When disagreements occur, the problems will be solved by discussion or consultation with a third author.

### 4.1 Statistical analysis

RevMan 5.3 Software will be utilized for the data synthesize and data analysis. Continuous data (such as depression, anxiety) will be summarized using standardized mean difference or mean difference and 95% confidence intervals (CIs). Binary data (such as incidence of adverse reactions) will be calculated using risk ratio and 95% CIs. Statistical heterogeneity will be evaluated by *I*^*2*^ statistic test. When *I*^*2*^ ≤ 50%, reasonable heterogeneity will be considered, and a fixed-effects model will be exerted, while when *I*^*2*^ > 50%, substantial heterogeneity will be considered, and a random-effects model will be presented. If sufficient homogeneity among included studies is identified, we will undertake a meta-analysis based on the similar characteristics of study and patient, interventions, comparators, and outcome measurements. On the other hand, we will explore subgroup analysis to detect the possible resources of significant heterogeneity. In addition, we will carry out a descriptive analysis by reporting written commentary to elaborate study findings.

### 4.2 Subgroup analysis

We will carry out subgroup analysis to identify potential sources of heterogeneity according to the characteristics of study and patient, details of interventions and controls, and outcome indicators.

### 4.3 Sensitivity analysis

We will undertake sensitivity analysis to check the robustness and stability of study findings by removing trials with high risk of bias.

### 4.4 Reporting bias

A Funnel plot and Eggers regression test will be generated to assess reporting bias when sufficient trials are included (normally over 10 trials).^[26,27]^

## 5 Grading the quality of evidence

Two authors will independently assess the quality of evidence for each outcome using Grading of Recommendations Assessment Development and Evaluation.^[28]^ Any different views will be solved by a third author via discussion.

## 6 Ethics and dissemination

Since this study will not utilize individual patient data, thus, no ethical approval is needed. This study will be published on a peer-reviewed journal or a conference meeting.

## 7 Discussion

With the advances in diagnosis, surgical management, perioperative care, the mortality and morbidity of EC has decreased substantially after operation. However, its surgery is still associated with a variety of disorders, such as psychological issues. Although studies suggest that HQNC can relieve depression, anxiety, and stress in patients with ECPP, their results are inconsistent.^[18–21]^ In addition, no systematic review specifically addressed this topic. Therefore, this study will firstly explore the effects of HQNC on psychological disorder in patients with ECPP systematically and comprehensively. The results of this study will provide evidence-based medicine proof of HQNC for the management of psychological disorder in patients with ECPP. It will benefit patients and clinical practice, as well as future studies.

## Author contributions

**Conceptualization:** Xiu-yu Liu, Dan Zhao, Hong-mei Zhang.

**Data curation:** Chuan-hua Jiao, Dan Zhao, Yan Chen, Hong-mei Zhang.

**Formal analysis:** Xiu-yu Liu, Chuan-hua Jiao, Hong-mei Zhang.

**Investigation:** Hong-mei Zhang.

**Project administration:** Hong-mei Zhang.

**Resources:** Xiu-yu Liu, Chuan-hua Jiao, Dan Zhao, Yan Chen.

**Software:** Xiu-yu Liu, Chuan-hua Jiao, Dan Zhao, Yan Chen.

**Supervision:** Hong-mei Zhang.

**Validation:** Xiu-yu Liu, Chuan-hua Jiao, Dan Zhao, Yan Chen, Hong-mei Zhang.

**Visualization:** Xiu-yu Liu, Chuan-hua Jiao, Yan Chen, Hong-mei Zhang.

**Writing – original draft:** Xiu-yu Liu, Chuan-hua Jiao, Dan Zhao, Yan Chen, Hong-mei Zhang.

**Writing – review & editing:** Xiu-yu Liu, Dan Zhao, Hong-mei Zhang.

## References

[1] Xu QL Li H Zhu YJ The treatments and postoperative complications of esophageal cancer: a review. J Cardiothorac Surg 2020;15:163. 32631428 10.1186/s13019-020-01202-2 PMC7336460

[2] Kim JA Shah PM Screening and prevention strategies and endoscopic management of early esophageal cancer. Chin Clin Oncol 2017;6:50. 29129090 10.21037/cco.2017.09.05

[3] Gao QY Fang JY Early esophageal cancer screening in China. Best Pract Res Clin Gastroenterol 2015;29:885–93. 26651250 10.1016/j.bpg.2015.09.018

[4] Moaven O Wang TN Combined modality therapy for management of esophageal cancer: current approach based on experiences from East and West. Surg Clin North Am 2019;99:479–99. 31047037 10.1016/j.suc.2019.02.004

[5] Bray F Ferlay J Soerjomataram I Global cancer statistics 2018: GLOBOCAN estimates of incidence and mortality worldwide for 36 cancers in 185 countries. CA Cancer J Clin 2018;68:394–424. 30207593 10.3322/caac.21492

[6] Chen W Zheng R Baade PD Cancer statistics in China, 2015. CA Cancer J Clin 2016;66:115–32. 26808342 10.3322/caac.21338

[7] Pachella LA Knippel S Symptom management for patients with esophageal cancer after esophagectomy. J Adv Pract Oncol 2016;7:741–7. 29670809 PMC5902153

[8] Sharma S Walsh D Symptom management in esophageal cancer. Chest Surg Clin N Am 1994;4:369–83. 8050000

[9] Wang Y Shi J Du L Health-related quality of life in patients with esophageal cancer or precancerous lesions assessed by EQ-5D: A multicenter cross-sectional study. Thorac Cancer 2020;11:1076–89. 32130756 10.1111/1759-7714.13368 PMC7113059

[10] Backemar L Johar A Wikman A The influence of comorbidity on health-related quality of life after esophageal cancer surgery. Ann Surg Oncol 2020;27:2637–45. 32162078 10.1245/s10434-020-08303-1 PMC7334248

[11] Mariette C Markar SR Dabakuyo-Yonli TS Hybrid minimally invasive esophagectomy for esophageal cancer. N Engl J Med 2019;380:152–62. 30625052 10.1056/NEJMoa1805101

[12] Petropoulos K Macheras A Liakakos T Minimally invasive esophagectomy for esophageal cancer: techniques and outcomes. Chirurgia (Bucur) 2015;110:99–108. 26011830

[13] McLaren PJ Dolan JP Esophagectomy as a treatment consideration for early-stage esophageal cancer and high-grade dysplasia. J Laparoendosc Adv Surg Tech A 2016;26:757–62. 27541368 10.1089/lap.2016.29010.pjm

[14] Hellstadius Y Lagergren J Zylstra J Prevalence and predictors of anxiety and depression among esophageal cancer patients prior to surgery. Dis Esophagus 2016;29:1128–34. 26542282 10.1111/dote.12437

[15] Abe T Hosoi T Kawai R Perioperative enteral supplementation with glutamine, fiber, and oligosaccharide reduces early postoperative surgical stress following esophagectomy for esophageal cancer. Esophagus 2019;16:63–70. 30030739 10.1007/s10388-018-0630-z

[16] Inoue T Ito S Ando M Changes in exercise capacity, muscle strength, and health-related quality of life in esophageal cancer patients undergoing esophagectomy. BMC Sports Sci Med Rehabil 2016;8:34. 27822378 10.1186/s13102-016-0060-y PMC5093971

[17] Takeshita N Kanda N Fukunaga T Esophageal intramural pseudodiverticulosis of the residual esophagus after esophagectomy for esophageal cancer. World J Gastroenterol 2015;21:9223–7. 26290650 10.3748/wjg.v21.i30.9223 PMC4533055

[18] Ma CH Zhang SX Niu WY The impact of nursing intervention on perioperative complications in patients undergoing radical esophageal cancer surgery. Chin Med Guide 2016;14:276–7.

[19] Chi XL Wang YF Zhou HX The impact of standardized nursing intervention on perioperative complications of patients undergoing esophageal cancer surgery. Dig World Latest Med Inform 2015;15:244.

[20] Gu X Zhao W Mi LL The effect of nursing intervention on the perioperative mental state of patients with esophageal cancer and diabetes. Hebei Med 2013;35:3655–6.

[21] Li XQ Geng HJ The effect of psychological intervention on the perioperative psychological status of patients with esophageal cancer. Chin Behav Med Sci 2005;6:529.

[22] Moher D Shamseer L Clarke M Preferred reporting items for systematic review and meta-analysis protocols (PRISMA-P) 2015 statement. Syst Rev 2015;4:1. 25554246 10.1186/2046-4053-4-1 PMC4320440

[23] Shamseer L Moher D Clarke M Preferred reporting items for systematic review and meta-analysis protocols (PRISMA-P) 2015 elaboration and explanation. BMJ 2015;350:g7647. 25555855 10.1136/bmj.g7647

[24] Jørgensen L Paludan-Müller AS Laursen DRT Evaluation of the Cochrane tool for assessing risk of bias in randomized clinical trials: overview of published comments and analysis of user practice in Cochrane and non-Cochrane reviews. Syst Rev 2016;5:80. 27160280 10.1186/s13643-016-0259-8 PMC4862216

[25] Stang A Critical evaluation of the Newcastle-Ottawa scale for the assessment of the quality of nonrandomized studies in meta-analyses. Eur J Epidemiol 2010;25:603–5. 20652370 10.1007/s10654-010-9491-z

[26] Sutton AJ Duval SJ Tweedie RL Empirical assessment of effect of publication bias on meta-analyses. BMJ 2000;320:1574–7. 10845965 10.1136/bmj.320.7249.1574 PMC27401

[27] Egger M Davey Smith G Schneider M Bias in meta-analysis detected by a simple, graphical test. BMJ 1997;315:629–34. 9310563 10.1136/bmj.315.7109.629 PMC2127453

[28] Mendoza C Kraemer P Herrera P Clinical guidelines using the GRADE system (Grading of Recommendations Assessment, Development and Evaluation). Rev Med Chil 2017;145:1463–70. 29664529 10.4067/s0034-98872017001101463

